# Changes in Childhood Body‐Mass Index and Risk of Venous Thromboembolism in Adulthood

**DOI:** 10.1161/JAHA.118.011407

**Published:** 2019-03-15

**Authors:** Jens Sundbøll, Lars Ängquist, Kasper Adelborg, Line Klingen Gjærde, Anne Ording, Thorkild I. A. Sørensen, Jennifer L. Baker, Henrik Toft Sørensen

**Affiliations:** ^1^ Department of Clinical Epidemiology Aarhus University Hospital Aarhus Denmark; ^2^ Department of Clinical Biochemistry Aarhus University Hospital Aarhus Denmark; ^3^ Center for Clinical Research and Prevention Bispebjerg and Frederiksberg Hospital Frederiksberg Denmark; ^4^ Novo Nordisk Foundation Center for Basic Metabolic Research (Section for Metabolic Genetics) University of Copenhagen Denmark; ^5^ Department of Public Health Section of Epidemiology Faculty of Health and Medical Sciences University of Copenhagen Denmark

**Keywords:** body mass index, deep venous thrombosis, pulmonary embolism, venous thromboembolism, Cardiovascular Disease, Obesity, Primary Prevention, Epidemiology

## Abstract

**Background:**

Childhood weight trajectories may influence cardiometabolic traits and thereby the risk of venous thromboembolism (VTE) later in life. We examined whether overweight and changes in weight status during childhood were associated with risk of VTE in adulthood.

**Methods and Results:**

We used Danish medical registries to conduct a population‐based cohort study of Danish schoolchildren aged 7 to 13 years and born during 1930‐1989. We calculated body‐mass index (BMI) z‐scores based on weight and height measurements. We estimated hazard ratios using Cox regressions to examine associations between changes in BMI z‐scores from 7 to 13 years of age and the subsequent risk of VTE. Among 313 998 children, 5007 girls and 5397 boys were diagnosed with VTE as adults. Compared with children with a normal BMI (25th to 75th percentile category) at both ages, children with a BMI persistently above the 75th percentile had a 1.30‐ to 1.50‐fold increased risk of VTE in adulthood. Children who experienced a BMI increase from the 25th to 75th or >75th to 90th percentile to a higher percentile category had a 1.35‐ to 1.70‐fold increased risk of adulthood VTE. Children whose BMI percentile category decreased between 7 and 13 years of age had a VTE risk similar to that of children with a persistently normal BMI.

**Conclusions:**

Risk of VTE in adulthood was higher in children with a persistently above‐average BMI. Whereas weight gain from 7 to 13 years of age additionally increased VTE risk, remission from overweight by 13 years of age completely reverted the risk.

**Clinical Perspective**

**What Is New?**

- Children with obesity have a cardiometabolic risk profile that may predispose them to venous thromboembolism later in life.
- We show that the risk of venous thromboembolism in adulthood was higher in children with a persistently above‐average body‐mass index.
- Whereas weight gain from 7 to 13 years of age additionally increased risk of venous thromboembolism, remission from overweight by 13 years of age completely reverted the risk.

**What Are the Clinical Implications?**

- Our observations suggest that childhood overweight may be an important modifiable risk factor for venous thromboembolism in adulthood.
- Because more children are becoming heavier at progressively younger ages, our results merit focus on helping children to attain and maintain appropriate weight to prevent cardiovascular disease in adulthood.

## Introduction

The global obesity epidemic affects ≈38% of the adult population and is spreading progressively to younger age groups.^1^ In high‐income countries, 20% of children aged 5 to 19 years are now classified as overweight.^2^ This prevalence among contemporary children reflects an almost 50% increase during the past 35 years^1^—a pattern that is expected to continue.^3^

Strong evidence suggests that factors acting throughout life contribute to the risk of cardiovascular disease. Evidence from observational studies indicates an inverse association between birth weight and the risk of myocardial infarction and stroke later in life.^4^, ^5^ This has been explained by fetal programming and in utero modification of genetic expression.^6^ During postnatal growth, childhood overweight and obesity are associated with both coronary artery disease^7^ and ischemic stroke in adulthood.^8^

It remains unclear if early growth patterns are also related to venous thromboembolism (VTE) in adulthood. Evidence of a relationship between atherosclerotic diseases and VTE is increasing.^9^, ^10^, ^11^, ^12^, ^13^ However, whether the association is causal or explained by shared risk factors is unknown. Recent publications have shown that conventional cardiovascular disease risk factors are not associated with increased VTE risk,^14^ and adjusting for such risk factors does not change the association between obesity and VTE in adults.^15^

To develop early preventive strategies, it is important to establish whether childhood overweight is a risk factor for VTE in adulthood and to understand whether this risk is reversible if normal weight is achieved before adolescence. Therefore, we investigated whether overweight and changes in weight status during childhood were associated with a later risk of VTE in a large cohort of children followed through adulthood.

## Methods

### Setting and Design

This population‐based cohort study included children born between 1930 and 1989 who took part in mandatory annual health examinations at public and private schools in Copenhagen, Denmark. The Danish Civil Registration System, which was established in 1968, records the civil and vital status for each Danish resident through a unique personal identification number assigned at birth or on immigration. The personal identification number allows for individual‐level linkage among an array of Danish registries.^16^

### Body‐Mass Index

The annual examinations included measurements of the children's weight and height when they were naked or wearing light clothing. The successive weight and height measures were noted on a personal health card including the child's name, date of birth, and birth weight as reported by the parents (since birth year 1936). These data have been stored digitally in the Copenhagen School Health Records Register.^17^ We successfully linked 88% of the records to the Civil Registration System. The main reason for linkage failure was death or emigration before 1968.^17^

We calculated each child's body‐mass index (BMI) from 7 through 13 years of age as the body weight in kilograms divided by the squared height in meters. Using data from 1955 to 1960, we created internal age‐ and sex‐specific BMI references. BMI z‐scores were calculated using the lambda‐mu‐sigma method.^18^ Z‐scores were estimated by interpolation or extrapolation in a ±12‐month time window unless measurements were recorded on the child's birthday.

### Venous Thromboembolism

We used the Danish National Patient Register, covering all Danish hospitals,^19^ to identify first‐time VTE events, collapsing deep venous thrombosis and pulmonary embolism, as these have been regarded as clinical manifestations of the same disease process. We included first‐time VTE events based on inpatient or outpatient admissions coded according to the *International Classification of Diseases*,* Eighth Revision* (*ICD‐8*) until the end of 1993 and *Tenth Revision* (*ICD‐10*) thereafter (Table S1). The Danish National Patient Register contains data on admission and discharge dates from all Danish nonpsychiatric hospitals since 1977 and from outpatient clinics since 1995.^19^

### Statistical Analysis

We followed all children who could be successfully linked to the Civil Registration System from the date of their 25th birthday (as VTE at younger ages is uncommon and likely related to congenital thrombogenic disorders) or from January 1, 1977 (when the Danish National Patient Register was established), whichever occurred later. The index date was defined as the date of commencement of follow‐up, which continued for each individual until a hospital admission or outpatient visit for VTE, death, emigration, or December 31, 2014, whichever occurred first.

Because the risk factors for the development of VTE differ for men and women,^20^, ^21^ we performed all analyses separately by sex. We calculated incidence rates for VTE and used Cox proportional‐hazard regression analyses with age as the underlying time scale to examine associations between BMI z‐scores and risk of VTE.

We performed categorical analyses based on the <10th, 10th to <25th, 25th to 75th (reference), >75th to 90th, and >90th percentiles of age‐ and sex‐specific BMI distributions. In addition to categorical analyses, we analyzed BMI z‐scores as a continuous variable. Potential nonlinearity in the associations between BMI z‐scores and VTE was tested against a restricted cubic spline with 4 knots.^22^ We found indications of nonlinearity for both sexes (majority of *P*<0.05). Therefore, we present these results using spline models. Because the patterns of associations were comparable at all ages, we only present the results for 7 and 13 years of age. Results for intermediate ages are shown in Table S2. To distinguish cases of VTE with and without known predisposing conditions, we analyzed the risks of provoked and unprovoked VTE separately, defining “provoked” VTE as the presence of a preceding malignancy (any time before the VTE diagnosis) or pregnancy/delivery, trauma/fracture, or surgery (within 90 days before the VTE diagnosis).^23^

In the cohort of individuals for whom birth‐weight data were available, we examined their association with VTE using categorical and spline models. In addition, we investigated in spline models the possible influence of birth weight by testing interactions between dichotomized birth weight and BMI on the risk of VTE using a likelihood ratio test and by adjusting for birth weight. All analyses were stratified by birth cohort (1930‐1939, 1940‐1949, 1950‐1959, 1960‐1969, 1970‐1979, and 1980‐1989), allowing the baseline hazard to differ for each birth cohort. In addition, we tested for potential interactions with birth cohorts in both categorical and spline models.

Proportional hazard assumptions were assessed by splitting the age‐at‐risk observation time into groups (based on age quartiles at VTE diagnosis) and performing corresponding interaction tests. *P*<0.05 held only for boys at 7 and 8 years of age. Thus, the proportional hazards assumption was not violated (Table S3).

To evaluate the impact of changes in BMI during childhood, we computed hazard ratios for VTE comparing children who changed BMI percentile categories between 7 and 13 years of age with children who maintained BMIs in the reference category (25th‐75th percentile) at both ages. This analysis was based on percentiles of age‐ and sex‐specific BMI distributions provided by the Centers for Disease Control and Prevention,^24^ where “overweight” is equivalent to the 90th percentile of BMI in our data.

Because deep venous thrombosis and pulmonary embolism were collapsed into a single entity (venous thromboembolism) in the main analysis, we performed a sensitivity analysis of categorical data separately for deep venous thrombosis and pulmonary embolism to test the robustness of this approach. In a sensitivity analysis we also calculated E‐values for selected representative estimates and the corresponding lower limit of the 95% CI for boys and girls aged 13 years with BMI z‐scores of 0.68 to 1.28. This allowed us to assess how strong an unmeasured confounder would have to be to explain away the observed exposure‐outcome association.^25^

All statistical analyses were performed using Stata statistical software version 14.2 (StataCorp LP, College Station, TX; www.stata.com). The study was approved by the Danish Data Protection Agency (record number 2012‐58‐0004). According to Danish legislation, no approval from an ethics committee or informed consent from patients is required for register‐based studies in Denmark.^26^ The data that support the findings of this study are available from the corresponding author on reasonable request.

## Results

**Figure 1:**
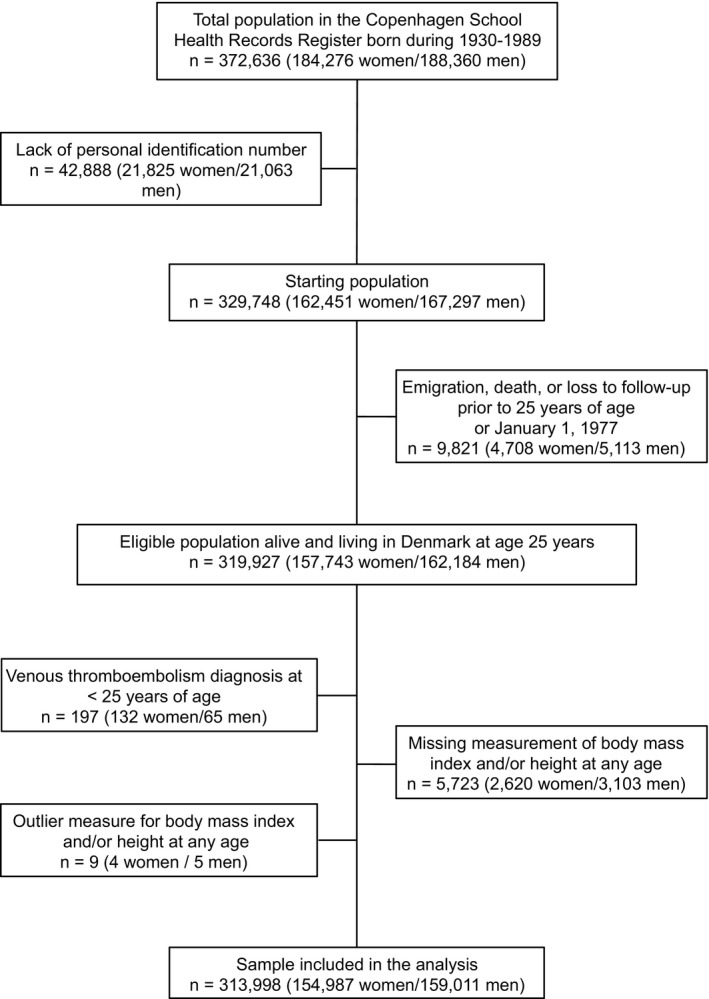
Inclusion of children in the study.

We identified a total study population of 372 636 children. After excluding individuals who did not have a personal identification number, individuals who had emigrated, died, or had VTE before age 25 years, or individuals with no BMI information, 313 998 children (154 987 girls and 159 011 boys) remained in the study (Figure 1). During 8 582 910 person‐years of follow‐up, 5007 girls and 5397 boys were diagnosed with first‐time VTE later in life. As expected, the incidence rate of VTE increased with advancing age and was moderately higher in men than in women (Figure S1).

### Childhood Body‐Mass Index and Risk of Venous Thromboembolism

Across sex and age, children with a BMI in the >75th to 90th percentile had a moderately increased risk of VTE in adulthood compared with children with a BMI in the 25th to 75th percentile, whereas a BMI above the 90th percentile was associated with a substantially increased risk (Table 1). For women with a childhood BMI above the 90th percentile at 7 or 13 years of age, the risk of VTE was 1.46‐fold and 1.61‐fold higher, respectively. For men with a childhood BMI above the 90th percentile at 7 or 13 years of age, the risk of VTE was 1.28‐fold and 1.50‐fold higher, respectively. The associations were comparable in analyses stratified by provoked and unprovoked VTE (Table S4). About one third of VTE cases were provoked; of these, 1852 were cancer related (1000 women and 852 men), 96 were pregnancy related (only women), 628 were fracture related (282 women and 346 men), and 1969 were surgery related (1018 women and 951 men). Of note, a single individual could have more than 1 provoking factor registered, and VTE events stratified by provoking factors exceeded the total number of unique VTE events. The results remained largely unchanged when we separately analyzed deep venous thrombosis and pulmonary embolism (Table S5).

**Table 1:** BMI Category and Risk of Venous Thromboembolism in Adulthood, by Age and Sex

Age, y	BMI z‐Score Category	BMI Percentile	Female			Male		
			BMI Equivalent (kg/m^2^)	No. of Events	HR (95% CI)	BMI Equivalent (kg/m^2^)	No. of Events	HR (95% CI)
7	−4.5 to −1.28	<10th	<13.8	440	0.97 (0.88‐1.07)	<14.0	419	0.84 (0.76‐0.93)
	−1.28 to −0.68	10th to <25th	13.8 to <14.5	671	0.94 (0.86‐1.02)	14.0‐<14.6	789	1.00 (0.92‐1.08)
	−0.68 to 0.68	25th to 75th	14.5 to 16.3	22 328	1.00 (reference)	14.6 to 16.3	2592	1.00 (reference)
	0.68 to 1.28	>75th to 90th	>16.3 to 17.3	714	1.12 (1.03‐1.22)	>16.3 to 17.2	788	1.10 (1.01‐1.19)
	1.28 to 4.50	>90th	>17.3	513	1.46 (1.32‐1.60)	>17.2	487	1.28 (1.16‐1.41)
13	−4.5 to −1.28	<10th	<15.7	379	0.90 (0.81‐1.00)	<15.6	399	0.84 (0.76‐0.93)
	−1.28 to −0.68	10th to <25th	15.7 to <16.7	618	0.90 (0.82‐0.98)	15.6‐<16.5	689	0.90 (0.83‐0.98)
	−0.68 to 0.68	25th to 75th	16.7 to 19.9	2323	1.00 (reference)	16.5 to 19.4	2555	1.00 (reference)
	0.68 to 1.28	>75th to 90th	>19.9 to 21.9	750	1.14 (1.05‐1.24)	>19.4 to 21.1	794	1.13 (1.04‐1.22)
	1.28 to 4.50	>90th	>21.9	578	1.61 (1.47‐1.76)	>21.1	559	1.50 (1.37‐1.65)

BMI indicates body‐mass index (kg/m^2^); HR, hazard ratio.

For boys aged 13 years (hazard ratio [HR] 1.13, 95% CI 1.04‐1.22), the E‐value was 1.51 for BMI z‐scores of 0.68 to 1.28 (E‐value for lower limit of 95% CI=1.24). For girls aged 13 years (HR 1.14, 95% CI 1.05‐1.24), the E‐value was 1.54 for BMI z‐scores of 0.68 to 1.28 (E‐value for lower limit of 95% CI was 1.28).

**Figure 2:**
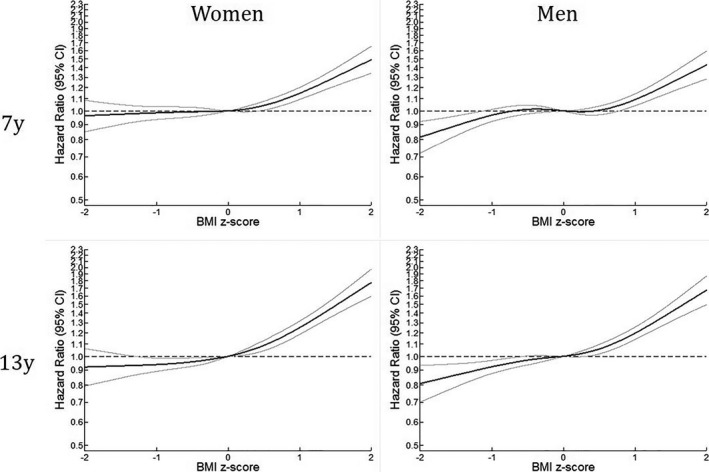
Restricted cubic spline models for the association between body‐mass index (BMI) at 7 and 13 years of age and venous thromboembolism in adulthood.

The results from the categorical analyses were reflected in the spline regression analyses (Figure 2) in which above‐average BMI z‐scores among boys at 7 or 13 years of age were positively associated with VTE in adulthood, with the strongest associations between BMI z‐scores at 13 years and subsequent VTE. The same pattern was observed in girls with above‐average BMI z‐scores. Below‐average childhood BMI values were associated with a marginally decreased risk of VTE, especially for boys with below‐average BMI at 13 years of age. We observed no birth cohort effects in the categorical or spline models (data not shown).

In the 117 418 girls and 122 538 boys who had data available on birth weight, 3736 girls and 3241 boys were diagnosed with VTE in adulthood. For these individuals, we found no association between low birth weight and VTE in adulthood (data not shown). Adjusting for birth weight did not change the associations between BMI z‐score and the risk of VTE in adulthood (Figure S2). In girls with birth weight z‐scores ≤0, we observed a moderately strengthened association between BMI at 7 and 13 years of age and risk of VTE in adulthood (Figure S3). In boys we observed no interactions between birth weight and BMI on VTE risk at any age.

### BMI Percentile Change During Childhood and Risk of VTE

The analyses of changes in BMI included 253 578 children (126 277 girls and 127 301 boys). Children with normal weight at 7 and 13 years of age (BMI within the 25th‐75th percentile defined by the Centers for Disease Control and Prevention) constituted the reference group in all comparisons. Children with a persistently above‐average BMI had a 1.28‐ to 2.43‐fold higher risk of VTE in adulthood (Table 2). Children who gained weight from the 25th to 75th percentile at 7 years of age to either the >75th to 90th or >90th percentile at 13 years of age had a 1.29‐ to 2.87‐fold higher risk of VTE during adulthood. Girls in either the >75th to 90th or >90th percentile at 7 years of age who then moved down 1 BMI category by 13 years of age had the same risk of VTE in adulthood as girls in the reference group. Boys who moved down from the >75th to 90th percentile at 7 years of age to the 25th to 75th percentile at 13 years of age also had the same risk as the reference group. In contrast, boys who moved down from the >90th percentile to the >75th to 90th percentile remained at a 2‐fold higher risk of VTE in adulthood.

**Table 2:** Hazard Ratios for the Association Between Different Patterns of Change in BMI Between 7 and 13 Years of Age and Diagnosis of VTE in Adulthood

BMI Percentile at Age 7 y^a^	BMI Percentile at Age 13 y^a^				
	<5th	5th to <25th	25th to 75th	>75th to 95th	>95th
Women					
<5th					
No. of cases/total no.^b^	78/2378	86/2440	28/778	2/20	0/0
Hazard ratio (95% CI)	0.99 (0.79‐1.24)	1.01 (0.81‐1.25)	1.05 (0.72‐1.52)	···	···
5th to <25th					
No. of cases/total no.^b^	76/2777	389/12 517	383/10 324	11/334	0/5
Hazard ratio (95% CI)	0.82 (0.66‐1.04)	0.85 (0.77‐0.95)	1.01 (0.90‐1.12)	1.04 (0.58‐1.88)	···
25th to 75th					
No. of cases/total no.^b^	10/545	364/11 843	1911/52 511	333/7829	7/100
Hazard ratio (95% CI)	0.61 (0.33‐1.13)	0.88 (0.78‐0.98)	1.00 (reference)	1.29 (1.14‐1.44)	2.87 (1.37‐6.03)
>75th to 95th					
No. of cases/total no.^b^	0/0	3/114	211/7033	353/8357	29/660
Hazard ratio (95% CI)	···	···	0.91 (0.79‐1.05)	1.35 (1.20‐1.51)	1.91 (1.32‐2.76)
>95th					
No. of cases/total no.^b^	0/0	0/0	2/77	29/787	27/516
Hazard ratio (95% CI)	···	···	···	1.36 (0.94‐1.96)	2.42 (1.65‐3.54)
Men					
<5th					
No. of cases/total no.^b^	73/3035	75/2218	29/544	1/26	0/0
Hazard ratio (95% CI)	0.68 (0.54‐0.86)	0.89 (0.71‐1.13)	1.54 (1.07‐2.23)	···	···
5th to <25th					
No. of cases/total no.^b^	114/3393	473/13 423	300/7048	16/268	1/2
Hazard ratio (95% CI)	0.91 (0.76‐1.10)	0.89 (0.81‐0.98)	1.11 (0.98‐1.25)	1.95 (1.20‐3.20)	···
25th to 75th					
No. of cases/total no.^b^	21/750	553/15 954	2025/51 362	271/5915	6/165
Hazard ratio (95% CI)	0.86 (0.56‐1.32)	0.88 (0.80‐0.97)	1.00 (reference)	1.35 (1.19‐1.54)	1.48 (0.66‐3.30)
>75th to 95th					
No. of cases/total no.^b^	0/2	6/168	353/8837	307/7459	26/666
Hazard ratio (95% CI)	···	1.02 (0.46‐2.27)	1.08 (0.97‐1.21)	1.28 (1.14‐1.45)	1.68 (1.14‐2.47)
>95th					
No. of cases/total no.^b^	0/0	0/0	4/73	35/662	28/614
Hazard ratio (95% CI)	···	···	···	2.01 (1.44‐2.80)	2.43 (1.67‐3.53)

Hazard ratios were not calculated for these BMI categories due to <5 available events. BMI indicates body‐mass index (kg/m^2^); VTE, venous thromboembolism.

a Based on age‐ and sex‐specific BMI distributions provided by the Centers for Disease Control and Prevention.

b Data in this row are the number of cases of venous thromboembolism diagnosed in adulthood and the total number of children with the given BMI pattern.

## Discussion

In this population‐based cohort study of 313 998 schoolchildren, above‐average BMI at 7 or 13 years of age was associated with an increased risk of VTE in adulthood. Children with persistently above‐average BMI at 7 and 13 years of age had an additionally increased risk of VTE in adulthood, whereas overweight children who normalized their BMI by 13 years of age had VTE risks comparable to those who were consistently normal weight. These results were consistent across sex and birth cohorts. The association between childhood BMI and risk of VTE in adulthood was moderately strengthened among girls with a birth‐weight z‐score ≤0. However, we found no association between low birth weight and VTE in adulthood. Moreover, adjusting for birth weight did not change the results appreciably.

Two previous case‐control studies^27^, ^28^ examined the risk of childhood‐onset VTE in obese children. Both studies involved a single institution, <100 VTE cases, and controls selected from patients admitted with other diseases. These studies reported a 2‐ to 3‐fold increased risk of VTE in obese children compared with normal‐weight children (odds ratio=2.1; 95% CI 1.1‐4.2^27^ and odds ratio=3.1; 95% CI 4.1‐7.0^28^)—similar to the risks described in adult cohorts.^29^, ^30^, ^31^ However, although obesity is an important and well‐established risk factor for VTE in both children and adults,^32^ previous studies of children^27^, ^28^ have not provided follow‐up into adulthood. A Danish cohort study of 6502 young adult men (median age 19 years) undergoing fitness examinations for army conscription examined overweight in relation to the risk of VTE. The study found that men with obesity (BMI≥30 kg/m^2^) had an almost 5‐fold increased risk of VTE later in life (HR 4.7; 95% CI 1.9‐11.9), whereas men who were overweight (BMI 25 to <30 kg/m^2^) had no increased risk compared with young men with BMI values between 18.5 and 25 kg/m^2^.^33^ In contrast, we found that BMI values even below the current childhood BMI classification of “overweight” used by the Centers for Disease Control and Prevention in the United States (equivalent to the 90th percentile of BMI in our study^24^) were associated with an increased risk of VTE in adulthood, pointing to an important association with even marginally increased BMI in childhood. This finding is notable and may indicate the need for increased clinical attention, especially considering the abundance of children in this category.

In our analyses of change in BMI percentile during childhood, we observed that children who were initially overweight but who had achieved normal weight before 13 years of age were not at increased risk of VTE in adulthood. This indicates that overweight in childhood may be a modifiable risk factor with the potential to prevent VTE. Contrary to these findings, weight loss has been associated with increased risk of provoked VTE in adults.^32^ This difference may exist because weight loss in adulthood can be an indicator of underlying disease, particularly cancer, whereas this factor only exceptionally may be the cause of weight loss in childhood.

Several putative mechanisms may be at the root of the observed associations. Shared determinants of later‐onset overweight and VTE may already exist in utero and involve the individual's specific gene pool and exposure to maternal lifestyle.^6^ Also, being overweight in childhood may correlate with overweight in adulthood and reflect a life‐long sedentary lifestyle that facilitates low‐flow conditions and thrombus formation in the venous system. Childhood overweight often continues into young adulthood.^34^ The derived risk for VTE is likely maintained, although the association between childhood BMI and middle to late adulthood BMI is weak.^34^ We could not examine the cardiometabolic trajectories because adult weight and cardiovascular risk factors have complex patterns over a lifetime, and adult weight preceding the occurrence of VTE could not be retrieved for this study.

An important strength of our study is the use of prospectively collected data on essentially every school child in the Copenhagen municipality from 1930 to 1989. Health examinations were mandatory at all public and private schools, which reduced selection bias stemming from differences in socioeconomic status or other possibly relevant factors. We were able to follow study participants over several decades of adulthood and had virtually no loss to follow‐up (<0.1%). The diagnosis of VTE in the Danish National Patient Register is valid for use in research, with a positive predictive value of ≈90%.^35^ Due to its acute onset, accompanying discomfort, and severe course, we assume that VTE rarely remains undetected. Thus, the outcome accuracy was favorable, and the likelihood of misclassification was low.

Several limitations must be considered in assessing our findings. We lacked information on socioeconomic status and smoking habits of the children and their parents. However, despite temporal changes in socioeconomic differences within birth cohorts and smoking habits, our results were consistent across all birth cohorts, indicating a minor role of such potential confounders. In support of this supposition, the derived E‐values indicating the strength of association with both the exposure and the outcome needed by an unmeasured confounder to potentially (as a maximum) explain away selected representative findings (HR 1.13‐1.14) were relatively large (E‐values: 1.51‐1.54) in comparison. This indicates that our findings are likely robust to effects of potential unmeasured and uncontrolled confounding. Finally, although our data are longitudinal, we were unable to clarify whether the associations were generated through metabolic effects of overweight in childhood or in adulthood—in the latter case because of retaining overweight status from childhood through adulthood.

In this study we demonstrated that, compared with normal‐weight children, above‐average BMI in childhood was associated with greater risk of VTE in adulthood. Furthermore, weight gain during childhood additionally increased this risk. Among overweight children who were able to normalize their weight before adolescence, the risk of VTE was the same as for children with normal weight throughout childhood. These observations suggest that childhood overweight may be an important modifiable risk factor for VTE in adulthood.

## Author Contributions

Sørensen and Sørensen conceived the study idea. Sundbøll, Sørensen, Baker, and Sørensen designed the study. Sundbøll directed the analyses, which were carried out by Ängquist and Gjærde. All authors participated in the discussion and interpretation of the results. Sundbøll reviewed the literature, organized the writing, and wrote the initial drafts. All authors critically revised the manuscript for intellectual content and approved the final version.

## Sources of Funding

This work was supported by the Program for Clinical Research Infrastructure (PROCRIN) established by the Lundbeck Foundation and the Novo Nordisk Foundation; the Aarhus University Research Foundation; and the European Union's Horizon 2020 Research and Innovation Programme (DynaHEALTH) under grant agreement no. 633595. No funding sources had a role in the design, conduct, analysis, or reporting of the study.

## Disclosures

None.

## Supporting information


**Table S1.** Definition of Venous Thromboembolism and Provoked Venous Thromboembolism According to Codes in the *International Classifications of Diseases*,* Eighth* and *Tenth Revisions*
**Table S2.** Body‐Mass Index Category and Risk of Venous Thromboembolism in Adulthood for Children at Ages 8, 9, 10, 11, and 12 Years, by Age and Sex
**Table S3.** Test of the Proportional Hazards Assumption in the Association Between Body‐Mass Index and Venous Thromboembolism
**Table S4.** Body‐Mass Index Category in Childhood and Risk of Provoked and Unprovoked Venous Thromboembolism in Adulthood
**Table S5.** Body‐Mass Index Category and Risk of Deep Venous Thrombosis or Pulmonary Embolism in Adulthood, by Age and Sex
**Figure S1**. Incidence rates of venous thromboembolism in adulthood, by sex.
**Figure S2.** Birth weight–adjusted restricted cubic spline models for the association between body‐mass index at ages 7 and 13 years and venous thromboembolism in adulthood. The unadjusted association is shown in black, and the association adjusted for birth weight is shown in orange.
**Figure S3.** Childhood body‐mass index (BMI) and risk of venous thromboembolism by birth‐weight z‐score (below vs above 0).
