# Supplementary material for: Changes in Childhood Body‐Mass Index and Risk of Venous Thromboembolism in Adulthood
Source: J Am Heart Assoc. 2019 Mar 15;8(6):e011407. doi: 10.1161/JAHA.118.011407 (PMC6475038; doi:10.1161/JAHA.118.011407)

SUPPLEMENTAL MATERIAL

**Table S1. Definition of venous thromboembolism and provoked venous thromboembolism, according to codes in the *International Classifications of Diseases*, *Eighth and Tenth Revisions* (ICD-8 and ICD-10).**			
	**ICD-8**	**ICD-10**	**Other codes**
**Definition of venous thromboembolism.**			
Deep venous thrombosis	451.00, 451.08, 451.09	I80.1, I80.2, I80.3	
Pulmonary embolism	450.99	I26.0, I26.9	
**Definition of provoked venous thromboembolism**. We defined patients with 'provoked' venous thromboembolism as those with a hospital discharge diagnosis of malignancy (any time before the venous thromboembolism diagnosis) or pregnancy/delivery, trauma/fracture, or surgery (within 90 days preceding the date of the venous thromboembolism diagnosis).			
**Cancer** (including polycythemia vera and essential thrombocythemia), any previous diagnosis	140–209, 28710	C00–C99, D45, D473	
**Fracture/trauma** within previous 90 days	800–929, 950–959	S00–T14	
**Surgery** within previous 90 days			Previous Danish Classification up to 1996: 000000-99960; Nordic Medico-statistical Committee's Classification of Surgical Procedures after 1996: KA-KQ, KX, KY
**Pregnancy** within previous 90 days	630–680	O00–O99	

Table S2. Body-mass index category and risk of venous thromboembolism in adulthood for children at ages 8, 9, 10, 11, and 12 years, by age and sex.

			Women			Men		
**Age (y)**	**BMI z-score category**	**BMI percentile**	**BMI equivalent (kg/m^2)**	**No. of events**	**HR (95% CI)**	**BMI equivalent (kg/m^2)**	**No. of events**	**HR (95% CI)**
8	-4.5 to -1.28	<10th	<14.0	346	0.97 (0.87–1.08)	<14.2	298	0.77 (0.68–0.87)
	-1.28 to -0.68	10th–<25th	14.0–<14.7	663	0.91 (0.84–1.00)	14.2–<14.9	739	1.01 (0.93–1.09)
	-0.68 to 0.68	25th–75th	14.7–16.7	2615	1.00 (reference)	14.9–16.7	2939	1.00 (reference)
	0.68 to 1.28	>75th–90th	>16.7–17.9	672	1.15 (1.05–1.25)	>16.7–17.8	776	1.16 (1.07–1.25)
	1.28 to 4.5	>90th	>17.9	486	1.49 (1.35–1.64)	>17.8	415	1.39 (1.25–1.54)
9	-4.5 to -1.28	<10th	<14.2	310	0.88 (0.79–0.99)	<14.5	292	0.76 (0.67–0.85)
	-1.28 to -0.68	10th–<25th	14.2–<15.0	654	0.93 (0.85–1.0)	14.5–<15.2	728	0.98 (0.91–1.07)
	-0.68 to 0.68	25th–75th	15.0–17.3	2617	1.00 (reference)	15.2–17.2	2909	1.00 (reference)
	0.68 to 1.28	>75th–90th	>17.3–18.7	669	1.13 (1.04–1.23)	>17.2–18.4	773	1.23 (1.14–1.34)
	1.28 to 4.5	>90th	>18.7	498	1.48 (1.34–1.63)	>18.4	421	1.42 (1.28–1.58)
10	-4.5 to -1.28	<10th	<14.4	306	0.91 (0.81–1.03)	<14.7	304	0.77 (0.68–0.86)
	-1.28 to -0.68	10th–<25th	14.4–<15.3	622	0.88 (0.80–0.96)	14.7–<15.5	703	0.90 (0.83–0.98)
	-0.68 to 0.68	25th–75th	15.3–17.8	2619	1.00 (reference)	15.5–17.7	2966	1.00 (reference)
	0.68 to 1.28	>75th–90th	>17.8–19.4	683	1.14 (1.05–1.24)	>17.7–19.1	694	1.15 (1.05–1.24)
	1.28 to 4.5	>90th	>19.4	501	1.49 (1.35–1.64)	>19.1	438	1.43 (1.30–1.59)

11	-4.5 to -1.28	<10th	<14.7	297	0.91 (0.81–1.02)	<15.0	312	0.80 (0.71–0.90)
	-1.28 to -0.68	10th–<25th	14.7–<15.6	619	0.90 (0.82–0.98)	15.0–<15.8	726	0.91 (0.84–0.99)
	-0.68 to 0.68	25th–75th	15.6–18.4	2564	1.00 (reference)	15.8–18.2	2927	1.00 (reference)
	0.68 to 1.28	>75th–90th	>18.4–20.1	719	1.20 (1.11–1.31)	>18.2–19.8	690	1.16 (1.07–1.26)
	1.28 to 4.5	>90th	>20.1	508	1.55 (1.41–1.71)	>19.8	439	1.42 (1.29–1.58)
12	-4.5 to -1.28	<10th	<15.1	284	0.88 (0.78–0.99)	<15.3	312	0.83 (0.73–0.93)
	-1.28 to -0.68	10th–<25th	15.1–<16.1	608	0.91 (0.83–1.00)	15.3–<16.1	714	0.91 (0.84–0.99)
	-0.68 to 0.68	25th–75th	16.1–19.1	2524	1.00 (reference)	16.1–18.8	2885	1.00 (reference)
	0.68 to 1.28	>75th–90th	>19.1–21.0	755	1.22 (1.12–1.32)	>18.8–20.5	696	1.14 (1.05–1.24)
	1.28 to 4.5	>90th	>21.0	516	1.54 (1.4–1.70)	>20.5	470	1.47 (1.33–1.62)

BMI, body-mass index (kg/m^2); CI, confidence interval; HR, hazard ratio

Table S3. Test of the proportional hazards assumption in the association between body-mass index and venous thromboembolism*

	Categorical model		Restricted cubic spline (4 knots)	
	**Women**	**Men**	**Women**	**Men**
Age (y)	p-value	p-value	p-value	p-value
7	0.25	<0.001	0.12	0.01
8	0.05	0.04	0.14	0.04
9	0.43	0.35	0.35	0.15
10	0.56	0.18	0.51	0.16
11	0.46	0.10	0.76	0.11
12	0.15	0.52	0.30	0.12
13	0.15	0.45	0.25	0.06

*A p-value <0.05 indicates non-proportional hazards.

Table S4. Body-mass index category in childhood and risk of provoked and unprovoked venous thromboembolism in adulthood.

					Provoked VTE			Unprovoked VTE		
**Sex**	**Age (y)**	**BMI z-score category**	**BMI percentile**	**BMI equivalent (kg/m^2)**	**N**	**No. of events**	**HR (95% CI)**	**N**	**No. of events**	**HR (95% CI)**
**Women**	7	-4.5 to 1.28	<10th	<13.8	12,811	139	0.91 (0.76–1.08)	12,698	252	0.99 (0.87–1.13)
		-1.28 to -0.68	10th–<25th	13.8–<14.5	21,869	266	0.96 (0.83–1.09)	21,722	413	0.91 (0.82–1.02)
		-0.68 to 0.68	25th–75th	14.5–16.3	77,078	954	1.00 (reference)	76,478	1,554	1.00 (reference)
		0.68 to 1.28	>75th–90th	>16.3–17.3	18,960	221	1.05 (0.91–1.22)	18,770	411	1.17 (1.05–1.31)
		1.28 to 4.5	>90th	>17.3	12,577	161	1.38 (1.17–1.63)	12,443	295	1.46 (1.28–1.65)
	13	-4.5 to -1.28	<10th	<15.7	9,980	106	0.90 (0.73–1.10)	9,905	181	0.91 (0.78–1.06)
		-1.28 to -0.68	10th–<25th	15.7–<16.7	18,120	210	0.89 (0.77–1.03)	17,995	335	0.87 (0.77–0.98)
		-0.68 to 0.68	25th–75th	16.7–19.9	71,443	949	1.00 (reference)	70,854	1,538	1.00 (reference)
		0.68 to 1.28	>75th–90th	>19.9–21.9	20,321	257	1.02 (0.89–1.17)	20,071	507	1.23 (1.11–1.36)
		1.28 to 4.5	>90th	>21.9	12,984	205	1.54 (1.33–1.80)	12,829	360	1.58 (1.41–1.77)
**Men**	7	-4.5 to -1.28	<10th	<14.0	13,002	106	0.77 (0.63–0.94)	12,849	259	0.85 (0.75–0.97)
		-1.28 to -0.68	10th–<25th	14.0–<14.6	21,193	221	0.93 (0.80–1.08)	20,884	530	1.02 (0.93–1.13)
		-0.68 to 0.68	25th–75th	14.6–16.3	79,789	884	1.00 (reference)	78,759	1,914	1.00 (reference)
		0.68 to 1.28	>75th–90th	>16.3–17.2	21,005	238	1.13 (0.98–1.30)	20,737	506	1.09 (0.99–1.21)

		1.28 to 4.5	>90th	>17.2	12,210	124	1.30 (1.08–1.57)	12,041	293	1.35 (1.20–1.53)
	13	-4.5 to -1.28	<10th	<15.6	10,230	84	0.77 (0.61–0.96)	10,097	217	0.87 (0.75–1.00)
		-1.28 to -0.68	10th–<25th	15.6–<16.5	19,307	195	0.86 (0.74–1.01)	19,051	451	0.90 (0.81–0.99)
		-0.68 to 0.68	25th–75th	16.5–19.4	73,857	872	1.00 (reference)	72,802	1,927	1.00 (reference)
		0.68 to 1.28	>75th–90th	>19.4–21.1	18,874	229	1.14 (0.98–1.32)	18,572	531	1.16 (1.06–1.28)
		1.28 to 4.5	>90th	>21.1	11,848	156	1.59 (1.34–1.89)	11,670	334	1.43 (1.27–1.60)

BMI, body-mass index (kg/m^2); CI, confidence interval; HR, hazard ratio, VTE, venous thromboembolism

Table S5. Body-mass index category and risk of deep venous thrombosis (DVT) or pulmonary embolism (PE) in adulthood, by age and sex.

			Women					Men				
				DVT		PE			DVT		PE	
Age, years	BMI z-score category	BMI percentile	BMI equivalent (kg/m^2)	No. of events	HR (95% CI)	No. of events	HR (95% CI)	BMI equivalent (kg/m^2)	No. of events	HR (95% CI)	No. of events	HR (95% CI)
7	-4.5 to -1.28	$<10^{th}$	<13.8	240	0.95 (0.83-1.09)	151	1.00 (0.84-1.19)	<14.0	262	0.90 (0.79-1.02)	103	0.71 (0.58-0.87)
	-1.28 to -0.68	10th–$<25^{th}$	13.8–<14.5	450	0.99 (0.89-1.10)	229	0.82 (0.71-0.95)	14.0–<14.6	502	1.00 (0.91-1.11)	249	0.98 (0.86-1.13)
	-0.68 to 0.68	25th–75th	14.5–16.3	1555	1.00 (reference)	953	1.00 (reference)	14.6–16.3	1857	1.00 (reference)	941	1.00 (reference)
	0.68 to 1.28	$>75^{th}$–90th	>16.3–17.3	403	1.17 (1.04-1.30)	229	1.10 (0.95-1.27)	>16.3–17.2	520	1.17 (1.06-1.29)	224	1.01 (0.87-1.16)
	1.28 to 4.50	$>90^{th}$	>17.3	293	1.51 (1.33-1.71)	163	1.45 (1.22-1.71)	>17.2	291	1.42 (1.25-1.60)	126	1.28 (1.06-1.54)
13	-4.5 to -1.28	$<10^{th}$	<15.7	177	0.92 (0.78-1.07)	110	0.96 (0.79-1.17	<15.6	2017	0.87 (0.76-1.01)	94	0.81 (0.66-1.00)
	-1.28 to -0.68	10th–$<25^{th}$	15.7–<16.7	362	0.95 (0.85-1.07)	183	0.78 (0.66-0.91)	15.6–<16.5	437	0.90 (0.82-1.00)	209	0.86 (0.74-1.00)
	-0.68 to 0.68	25th–75th	16.7–19.9	1532	1.00 (reference)	955	1.00 (reference)	16.5–19.4	1861	1.00 (reference)	938	1.00 (reference)
	0.68 to 1.28	$>75^{th}$–90th	>19.9–21.9	491	1.19 (1.08-1.32)	273	1.08 (0.94-1.24)	>19.4–21.1	522	1.19 (1.08-1.32)	238	1.11 (0.97-1.28)
	1.28 to 4.50	$>90^{th}$	>21.9	362	1.65 (1.47-1.85)	203	1.57 (1.35-1.83)	>21.1	343	1.56 (1.39-1.75)	147	1.44 (1.21-1.72)

BMI, body-mass index (kg/m^2); CI, confidence interval; HR, hazard ratio

Figure S1. Incidence rates of venous thromboembolism in adulthood, by sex.

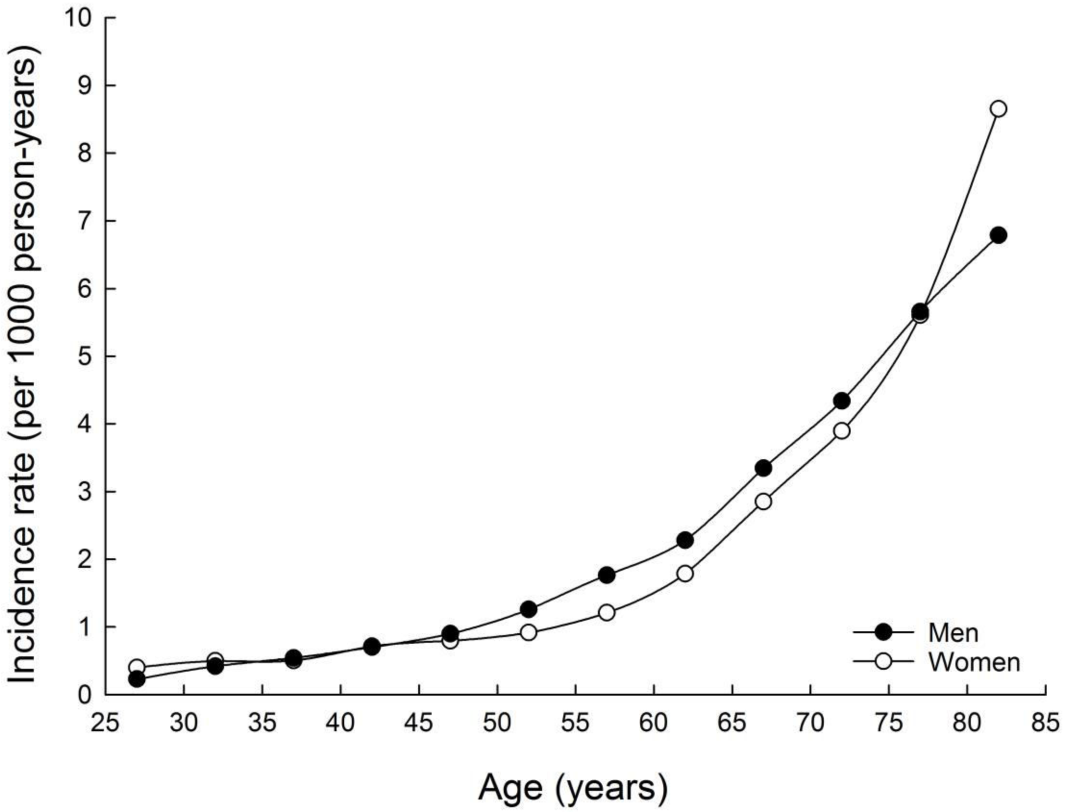

Figure S2. Birth-weight adjusted restricted cubic spline models for the association between body-mass index at ages 7 and 13 years and venous thromboembolism in adulthood. The unadjusted association is shown in black and the association adjusted for birth weight is shown in orange.

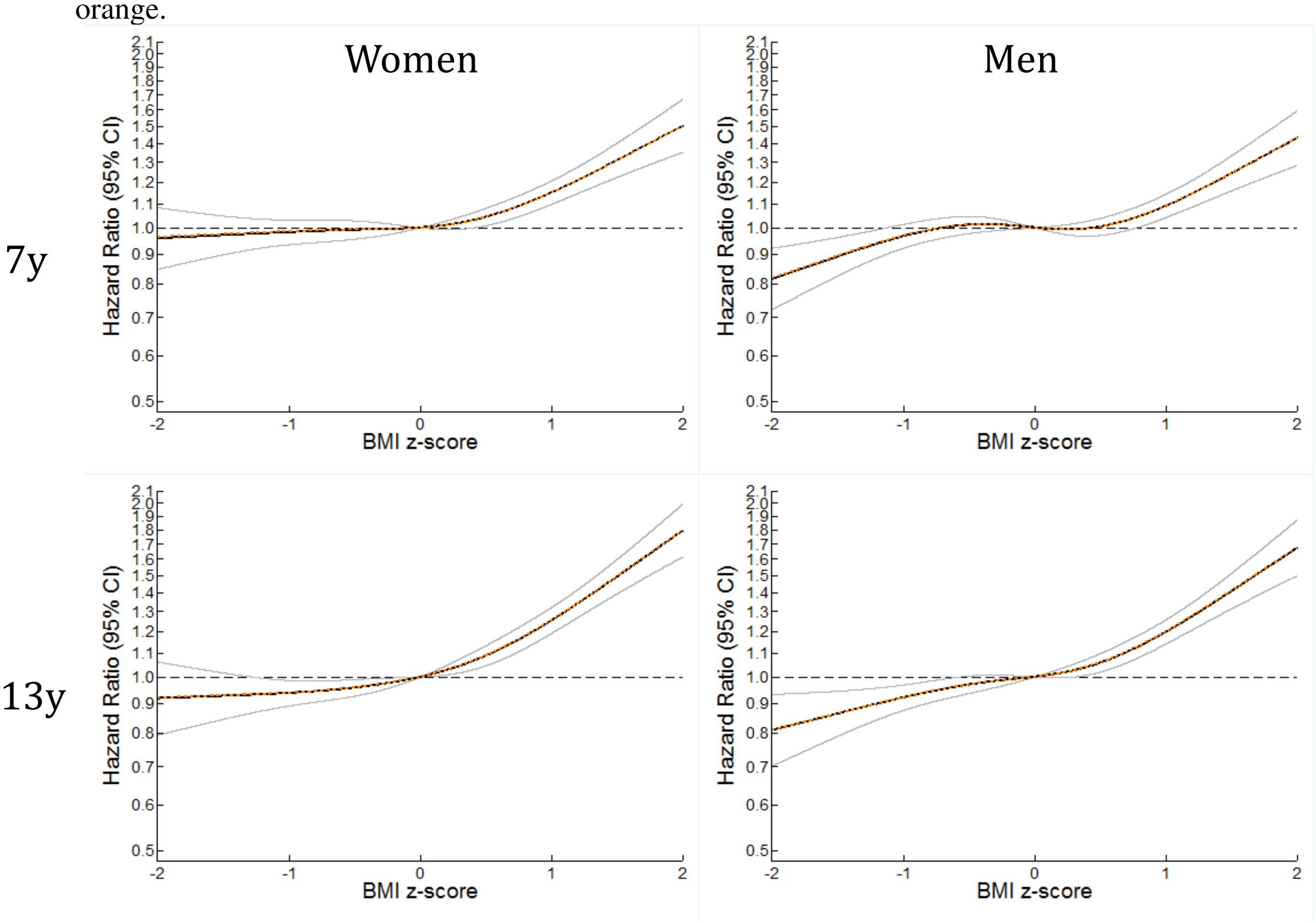

Figure S3. Childhood body-mass index (BMI) and risk of venous thromboembolism, by birth weight z-score (below *vs.* above 0).

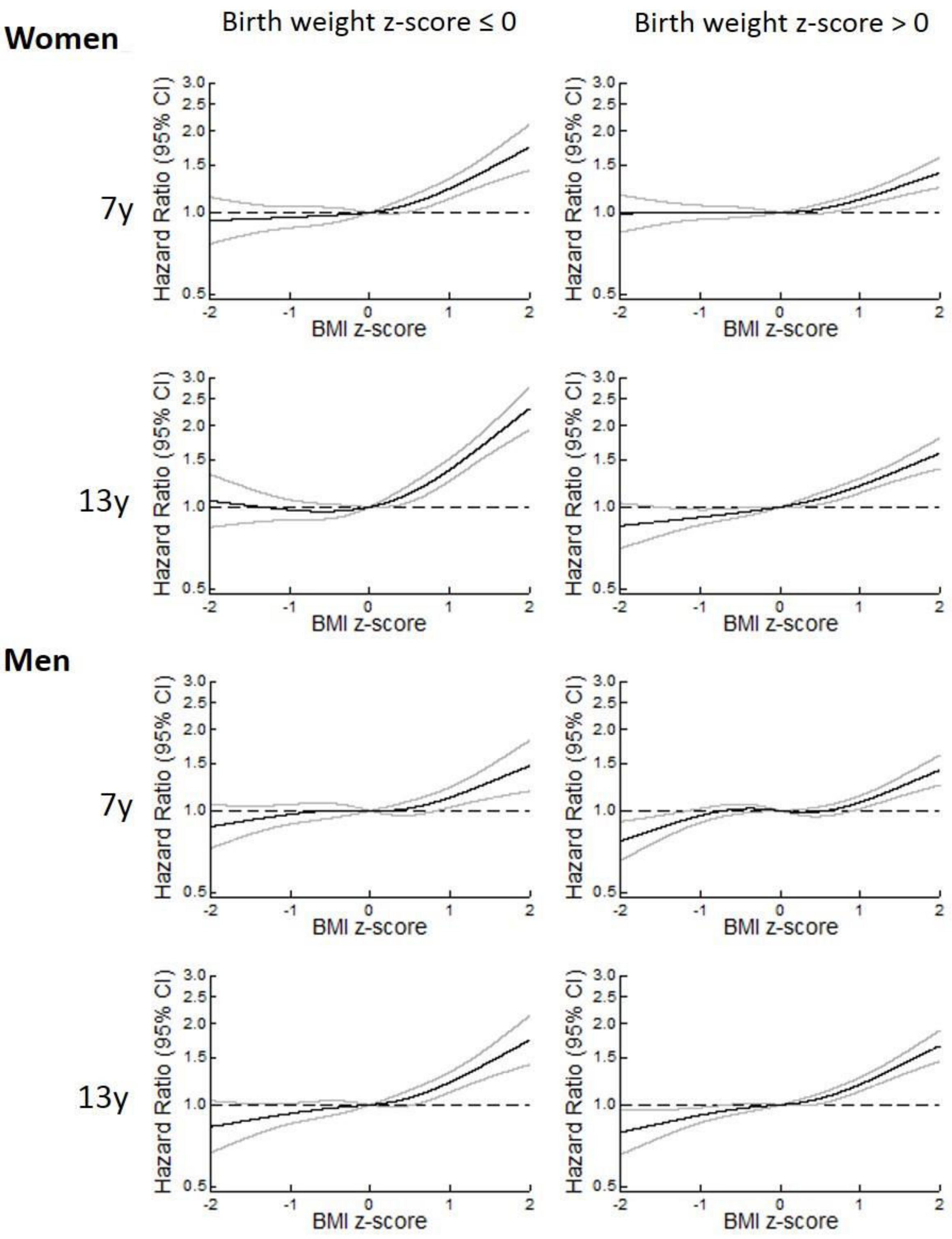

Supplement: Supplementary file 1 — Table S1. Definition of Venous Thromboembolism and Provoked Venous Thromboembolism According to Codes in the International Classifications of Diseases, Eighth and Tenth Revisions Table S2. Body‐Mass Index Category and Risk of Venous Thromboembolism in Adulthood for Children at Ages 8, 9, 10, 11, and 12 Years, by Age and Sex Table S3. Test of the Proportional Hazards Assumption in the Association Between Body‐Mass Index and Venous Thromboembolism Table S4. Body‐Mass Index Category in Childhood and Risk of Provoked and Unprovoked Venous Thromboembolism in Adulthood Table S5. Body‐Mass Index Category and Risk of Deep Venous Thrombosis or Pulmonary Embolism in Adulthood, by Age and Sex Figure S1. Incidence rates of venous thromboembolism in adulthood, by sex. Figure S2. Birth weight–adjusted restricted cubic spline models for the association between body‐mass index at ages 7 and 13 years and venous thromboembolism in adulthood. The unadjusted association is shown in black, and the association adjusted for birth weight is shown in orange. Figure S3. Childhood body‐mass index (BMI) and risk of venous thromboembolism by birth‐weight z‐score (below vs above 0). [file JAH3-8-e011407-s001.pdf]
